# Contact allergy in children with and without atopic dermatitis: An Italian multicentre study

**DOI:** 10.1111/cod.14130

**Published:** 2022-04-28

**Authors:** Domenico Bonamonte, Katharina Hansel, Paolo Romita, Anna Belloni Fortina, Giampiero Girolomoni, Gabriella Fabbrocini, Cataldo Patruno, Maddalena Napolitano, Annalisa Patrizi, Giuseppe Argenziano, Giuseppe Micali, Piergiacomo Calzavara Pinton, Caterina Foti, Luca Stingeni, Italian Society of Dermatology and Venereology, Pediatric Dermatology Group, Chiara Barlusconi, Francesco Bellinato, Stefano Caccavale, Giulia Calabrese, Francesca Caroppo, Aurora De Marco, Angela Filoni, Rossella Marietti, Maria Rita Nasca, Iria Neri, Donatella Schena, Marta Tramontana, Annalisa Vascellaro

**Affiliations:** ^1^ Section of Dermatology, Department of Biomedical Science and Human Oncology University of Bari “Aldo Moro” Bari Italy; ^2^ Section of Dermatology, Department of Medicine and Surgery University of Perugia Perugia Italy; ^3^ Unit of Pediatric Dermatology, Department of Medicine DIMED University of Padua Padua Italy; ^4^ Section of Dermatology, Department of Medicine University of Verona Verona Italy; ^5^ Section of Dermatology, Department of Clinical Medicine and Surgery University of Naples Federico II Italy; ^6^ Department of Health Sciences University Magna Graecia of Catanzaro Catanzaro Italy; ^7^ Department of Medicine and Health Sciences Vincenzo Tiberio University of Molise Campobasso Italy; ^8^ Unit of Dermatology IRCCS Azienda Ospedaliero Universitaria, University of Bologna Bologna Italy; ^9^ Dermatology Unit, Department of Mental and Physical Health and Preventive Medicine University of Campania Luigi Vanvitelli Naples Italy; ^10^ Dermatology Clinic University of Catania Catania Italy; ^11^ Dermatology Department University of Brescia Brescia Italy

**Keywords:** atopic dermatitis, children, contact allergy, patch testing

## Abstract

**Background:**

Contact allergy and atopic dermatitis (AD) are both common inflammatory T cell‐mediated diseases and many factors may influence the prevalence of contact allergy in AD patients. In children, their possible correlation was debated with conflicting results.

**Objectives:**

The present study aimed to assess the prevalence of contact sensitivity in children and to investigate the association with AD.

**Materials and methods:**

A retrospective multicentre study on children aged from 0 to 14 years patch tested between January 2017 and December 2018 was performed. Children were consecutively patch tested with the SIDAPA (Società Italiana Dermatologia Allergologica Professionale Ambientale) baseline series.

**Results:**

Among the 432 children investigated for contact allergy, 125 (28.9%) showed a positive reaction to at least one of the allergens tested, with a higher prevalence of positive patch test reactions in girls (32.3%) than in boys (25.0%). The most frequent contact allergens were nickel sulphate (10.2%), cobalt chloride (6.7%), methylisothiazolinone (3.7%), fragrance mix‐2 (3.2%), potassium dichromate (2.8%), fragrance mix‐1 (2.1%) and methylchloroisothiazolinone/methylisothiazolinone (2.1%). One‐hundred‐three children (23.8%) suffered from AD showing a higher prevalence of positive patch test (36.9%) compared to children without AD (26.4%).

**Conclusions:**

Despite the topic being still controversial, the present study suggests a consistent prevalence of contact allergy among children with higher sensitivity rate among children with AD than without AD.

## Graphical abstract

A total of 432 children aged from 0 to 14 years were patch tested. One hundred twenty‐five (28.9%) showed positive responses. AD children showed higher prevalence of positive patch test (36.9%) compared to children without AD (26.4%). Fragrance mix I and II and methylisothiazolinone were more frequently positive in children with than without AD.

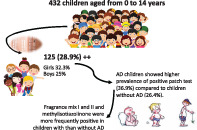

## 1 INTRODUCTION

In the past, contact allergy was considered rare and probably underestimated in children due to the immaturity of the childhood immune system and the low frequency of exposure to contact sensitizers in paediatric population.^1^ In the last decade, few large‐scale studies on childhood contact allergy published in Europe and North America showed that contact sensitization in children is more common than previously thought with rates of sensitization ranging from 36.2% to 62.3%.^2^, ^3^, ^4^, ^5^

Sensitization to contact allergens can occur as early as infancy^6^ and patch testing is the gold standard to diagnose contact allergy in children.^2^, ^4^, ^6^, ^7^ The prevalence of childhood contact allergy is influenced by several factors (new fashion in body piercing, use of personal care products, sports, and hobbies) and the most frequent sources of contact allergy in children are metals (nickel sulphate, potassium dichromate, cobalt chloride), fragrances, topical antibiotics (neomycin sulphate and bacitracin), emollients and emulsifiers (propylene glycol), and surfactants (cocamidopropyl betaine).^1^ Besides metals, the most frequent contact allergens in all ages, contact allergens in children vary according to age: neomycin sulphate, methylchloroisothiazolinone/methylisothiazolinone (MCI/MI) and lanolin alcohols in 1–5 years old children, neomycin sulphate, *Myroxylon pereira*, and fragrance mix‐1 in 6–12 years old children, and *p*‐phenylenediamine, fragrance mix‐1, and MCI/MI in 13–16 years old children.^4^

Nowadays, the role of atopic dermatitis (AD) as a favouring factor for contact allergy is debated and conflicting data have been reported in literature with a high prevalence range (from 27.0% to 95.6%) depending on study designs.^8^, ^9^, ^10^, ^11^, ^12^, ^13^, ^14^, ^15^, ^16^, ^17^, ^18^, ^19^, ^20^, ^21^, ^22^ In the past, murine and human models suggested that AD could be protective against contact allergy^23^, ^24^ since a prevalent Th2 response may lead to a relative cell‐mediated immune deficiency.^25^ Recent literature data have demonstrated increased risk of contact allergy in patients with AD due to multiple factors, such as a constitutionally reduced skin barrier function,^26^ also damaged by the frequent use of irritant chemicals,^27^ the continuous local use of emollients and anti‐inflammatory ointments with potential sensitizing properties,^28^ and a reduced heterogeneity of the AD skin microbiome.^29^, ^30^ Moreover, the relationship between contact allergy and AD seems to be even more complex as different immune pathways (Th1, Th2, and even Th17 mediated ones) may be shared by both entities.^31^ The most frequently reported contact allergens in AD are metals (nickel sulphate, cobalt chloride, and potassium dichromate), lanolin alcohol, neomycin sulphate, formaldehyde, sesquiterpene lactone mix, Compositae mix, and fragrances.^10^, ^12^, ^13^, ^14^, ^15^, ^16^, ^17^, ^18^, ^19^, ^20^ Considering that also some “hypoallergenic” personal care products can contain powerful contact allergens,^28^, ^32^ lanolin and fragrances were recently reported as the most common allergens in AD children by European^22^ and North American^33^ researchers.

In this multicentric retrospective study, we analysed the prevalence of contact sensitivity in children aged from 0 to 14 years undergoing patch testing for eczematous dermatitis, also highlighting the possible correlations with gender and atopic dermatitis.

## 2 MATERIALS AND METHODS

Data from 11 dermatological referral centres homogeneously distributed in Northern, Central, and Southern Italy were collected during a 2‐year period between January 2017 and December 2018. In this multicentric retrospective study, children with eczematous dermatitis aged from 0 to 14 years were all consecutively patch tested with the SIDAPA (Società Italiana Dermatologia Allergologica Professionale Ambientale) baseline series.^34^ Patients with acute eczematous lesions underwent patch testing 2 weeks after the resolution of lesions treated with topical corticosteroid. Patch tests were applied on the patient's back with the Haye's Test Chambers (Haye's Service B.V.) on Soffix tape (Artsana). Allergens were occluded for 2 days in all children and were provided by FIRMADiagent. The patch test readings were performed at Day (D)2 and D4 and patients were asked to return if there were new late reactions beyond D4. The score of positive patch test reaction was recorded as + (mild), ++ (strong), and +++ (extreme) at each reading time; irritant and doubtful responses were recorded as negative results.^35^ Being the study retrospective, it was not possible to establish the relevance of all positive patch test results. Patch test results were analysed according to five age groups (0 to 3, ≥3 to <6, ≥6 to <9, ≥9 to <12, ≥12 to <15 years) and the presence of AD at the time of testing. The diagnosis of atopic dermatitis was made according to Hanifin and Rajka criteria.^36^

The study protocol was approved by the ethics committees of the participating centres. Signed informed consent was obtained from patients' parents. Differences of paired discrete data were tested by Fisher's exact test and were used to analyse categorical variables. All statistical analyses were performed using IBM‐SPSS version 26.0 (IBM Corp., 2019) and using R software, version 4.0.3. In all analyses, a two‐sided *p* value ≤0.05 with Bonferroni correction was considered significant.

## 3 RESULTS

Among the 432 children (200 boys, 46.3%; 232 girls, 53.7%; mean age: 10.4 years), 125 (28.9%) showed a positive reaction to at least one of the patch‐tested contact allergens, all in D2–D4 without any late reaction beyond D4 (Table 1). In particular, 50 of them (40.0%) were boys and 75 (60.0%) girls. The prevalence of contact sensitivity was 25.0% and 32.3% in boys and girls, respectively. The total number of positive patch test reactions was 185 with a mean number of 1.5 reactions/patient, irrespectively of gender (75/50 in boys, 110/75 in girls). Eighty‐eight of one hundred twenty‐five sensitized children (70.4%) were mono‐sensitized, 15 (12.0%) showed two positive reactions, and 22 (17.6%) had at least three positive reactions.

**TABLE 1:** Demographics and patch test results according to gender and atopic dermatitis in 432 patch tested children

	No. of children (%)	Atopic dermatitis (%)	Boys (%)	Girls (%)	Children with at least one positive patch test result (%)	Boys (%)	Girls (%)
Total	432	103 (23.8) CI: 20.1–28.1	200 (46.3) CI: 41.6–51.0	232 (53.7) CI: 50.0–58.4	125 (28.9) CI: 24.9–33.4	50 (25.0) CI: 19.5–31.4	75 (32.3) CI: 26.6–38.6
Age groups (years)							
0 to <3	59 (13.7)	9 (15.3)	28 (47.5)	31 (52.5)	13 (22.0)	4 (14.3)	9 (29.0)
≥3 to <6	87 (20.1)	24 (27.6)	43 (49.4)	44 (50.6)	18 (20.7)	6 (14.0)	12 (27.3)
≥6 to <9	65 (15.0)	23 (35.4)	26 (40.0)	39 (60.0)	18 (27.7)	10 (38.5)	8 (20.5)
≥9 to <12	113 (26.2)	25 (22.1)	57 (50.4)	56 (49.6)	36 (31.9)	14 (24.6)	22 (39.3)
≥12 to <15	108 (25.0)	22 (20.4)	46 (42.6)	62 (57.4)	40 (37.0)	16 (34.8)	24 (38.7)
Atopic dermatitis							
Yes		103 (23.8) CI: 20.1–28.1	49 (47.6) CI: 38.2–57.1	54 (52.4) CI: 42.9–61.8	38 (36.9) CI: 28.2–46.5	16 (32.7) CI: 21.2–46.6	22 (40.7) CI: 28.7–54.0
No		329 (76.2) CI: 71.9–79.9	151 (45.9) CI: 40.6–51.3	178 (54.1) CI: 48.7–59.4	87 (26.4) CI: 22.0–31.5	34 (22.5) CI: 16.6–30.0	53 (29.8) CI: 23.5–36.9

According to age groups, the highest prevalence (37.0%) of positive patch test results was observed in the oldest age group (12–14 years), with a decreasing trend in the 9–11 and 6–8 years age groups (31.9% and 27.7%, respectively), while the lowest prevalence (20.7%) was reported in the 3–5 years age group. The higher prevalence of positive patch test reactions in girls than in boys was confirmed in all of the age groups, except for the 6–8 years age group, where this prevalence was higher in boys than in girls (15.4% vs. 12.3%).

Atopic comorbidities (allergic rhinitis, conjunctivitis, and asthma) were present in 258 of tested children (59.7%). In particular, 103 (23.8%) suffered from AD. Among these, 38 (36.9%) presented with at least 1 positive patch test. These data resulted higher than that observed in the remaining 329 children without AD, where 87 (26.4%) presented with at least one positive patch test reaction. The mean number of positive patch test reactions in the 38 children with AD and in the 87 children without AD was 1.4 and 1.5 reactions/patient, respectively. Considering gender, girls resulted more frequently contact sensitized than boys both in children with (40.7% vs. 32.7%, respectively) and without (29.8% vs. 22.5%, respectively) AD.

Globally, the most frequent contact allergens were nickel sulphate, cobalt chloride, MI, fragrance mix‐2, potassium dichromate, fragrance mix‐1, MCI/MI, neomycin sulphate, and dimethyl propylamine (Table 2). Considering gender, the allergens with considerably higher prevalence in girls than in boys were nickel sulphate (12.1% vs. 8.0%), cobalt chloride (5.5% vs. 7.8%), MI (4.8% vs. 2.5%), fragrance mix‐2 (4.3% vs. 2.0%), and potassium dichromate (2.6% vs. 0.3%). The presence of AD correlated to a higher prevalence of contact allergy for the eight most frequently positive contact allergens (nickel sulphate, cobalt chloride, MI, fragrance mix‐2, potassium dichromate, fragrance mix‐1, MCI/MI, neomycin sulphate), with the highest prevalence differences for fragrance mix‐1 (5.8% vs. 0.9%; *p* = 0.004293), followed by fragrance mix‐2 (5.8% vs. 2.4%), MI (5.8% vs. 3.0%), and nickel sulphate (11.7% vs. 9.7%). For the less frequently positive contact allergens (dimethyl propylamine, colophony, textile dye mix, formaldehyde, p‐phenylenediamine, p‐tert‐butylphenol formaldehyde resin, benzocaine, epoxy resin, hydrocortisone‐21‐acetate, thiuram mix, N‐isopropyl‐N'‐phenyl‐p‐phenylenediamine, mercaptobenzothiazole, and 2‐hydroxyethyl methacrylate), the prevalence of positive reactions resulted slightly higher in children without AD than in those with AD.

**TABLE 2:** Positive contact allergens according to gender and atopic dermatitis

	Patch tested children: 432 (%)	Gender		Atopic dermatitis	
		Boys: 200 (%)	Girls: 232 (%)	Yes: 103 (%)	No: 329 (%)
Contact allergens					
Nickel sulphate (5% pet.)	44 (10.2) CI: 7.7–13.4	16 (8.0) CI: 5.0–12.6	28 (12.1) CI: 8.5–16.9	12 (11.7) CI: 6.8–19.3	32 (9.7) CI: 7.0–13.4
Cobalt chloride (1% pet.)	29 (6.7) CI: 4.7–9.5	11 (5.5) CI: 3.1–9.6	18 (7.8) CI: 5.0–11.9	8 (7.8) CI: 4.0–14.6	21 (6.4) CI: 4.2–9.6
Methylisothiazolinone (0.2% aq.)	16 (3.7) CI: 2.3–5.9	5 (2.5) CI: 1.1–5.7	11 (4.7) CI: 2.7–8.3	6 (5.8) CI: 2.7–12.1	10 (3.0) CI: 1.7–5.5
Fragrance mix‐2 (14% pet.)	14 (3.2) CI: 1.9–5.2	4 (2.0) CI: 0.8–5.0	10 (4.3) CI: 2.4–7.8	6 (5.8) CI: 2.7–12.1	8 (2.4) CI: 1.2–4.7
Potassium dichromate (0.5% pet.)	12 (2.8) CI: 1.6–4.8	6 (3.0) CI: 1.4–6.4	6 (2.6) CI: 1.2–5.5	4 (3.9) CI: 1.5–9.6	8 (2.4) CI: 1.2–4.7
Fragrance mix‐1 (8% pet.)	9 (2.1) CI: 1.1–3.9	4 (2.0) CI: 0.8–5.0	5 (2.2) CI: 0.9–4.9	6 (5.8) CI: 2.7–12.1	3 (0.9) CI: 0.3–2.6
Methylchloroisothiazolinone/methylisothiazolinone (0.02% aq.)	9 (2.1) CI: 1.1–3.9	4 (2.0) CI: 0.8–5.0	5 (2.2) CI: 0.9–4.9	3 (2.9) CI: 1.0–8.2	6 (1.8) CI: 0.8–3.9
Neomycin sulphate (20.0% pet.)	8 (1.9) CI: 0.9–3.6	3 (1.5) CI: 0.5–4.3	5 (2.2) CI: 0.9–4.9	3 (2.9) CI: 1.0–8.2	5 (1.5) CI: 0.7–3.5
Dimethyl propylamine (1.0% aq.)	6 (1.4) CI: 0.6–3.0	3 (1.5) CI: 0.5–4.3	3 (1.3) CI: 0.4–3.7	1 (1.0) CI: 0.2–5.3	5 (1.5) CI: 0.7–3.5
Colophony (20.0% pet.)	5 (1.2) CI: 0.5–2.7	2 (1.0) CI: 0.3–3.6	3 (1.3) CI: 0.4–3.7	0	5 (1.5) CI: 0.7–3.5
Textile dye mix (6.6% pet.)	5 (1.2) CI: 0.5–2.7	2 (1.0) CI: 0.3–3.6	3 (1.3) CI: 0.4–3.7	1 (1.0) CI: 0.2–5.3	4 (1.2) CI: 0.5–3.1
Formaldehyde (2.0% aq.)	4 (0.9) CI: 0.3–2.4	2 (1.0) CI: 0.3–3.6	2 (0.9) CI: 0.2–3.1	0	4 (1.2) CI: 0.5–3.1
*Myroxylon pereirae* (25.0% pet.)	4 (0.9) CI: 0.3–2.4	1 (0.5) CI: 0.01–2.8	3 (1.3) CI: 0.4–3.7	1 (1.0) CI: 0.2–5.3	3 (0.9) CI: 0.3–2.6
*p*‐Phenylenediamine (1.0% pet.)	4 (0.9) CI: 0.3–2.4	1 (0.5) CI: 0.01–2.8	3 (1.3) CI: 0.4–3.7	0	4 (1.2) CI: 0.5–3.1
*p*‐*tert*‐Butylphenol formaldehyde resin (1.0% pet.)	4 (0.9) CI: 0.3–2.4	3 (1.5) CI: 0.5–4.3	1 (0.4) CI: 0.08–2.4	0	4 (1.2) CI: 0.5–3.1
Benzocaine (5.0% pet.)	2 (0.5) CI: 0.1–1.7	1 (0.5) CI: 0.01–2.8	1 (0.4) CI: 0.08–2.4	0	2 (0.6) CI: 0.2–2.2
Epoxy resin (1.0% pet.)	2 (0.5) CI: 0.1–1.7	2 (1.0) CI: 0.3–3.6	0	0	2 (0.6) CI: 0.2–2.2
Paraben mix (16.0% pet.)	2 (0.5) CI: 0.1–1.7	1 (0.5) CI: 0.01–2.8	1 (0.4) CI: 0.08–2.4	1 (1.0) CI: 0.2–5.3	1 (0.3) CI: 0.05–1.7
Hydrocortisone‐21‐acetate (1.0% pet.)	2 (0.5) CI: 0.1–1.7	2 (1.0) CI: 0.3–3.6	0	0	2 (0.6) CI: 0.2–2.2
Thiuram mix (1% pet.)	1 (0.2) CI: 0.04–1.3	0	1 (0.4) CI: 0.08–2.4	0	1 (0.3) CI: 0.05–1.7
N‐isopropyl‐N'‐phenyl‐*p*‐phenylenediamine (0.1% pet.)	1 (0.2) CI: 0.04–1.3	1 (0.5) CI: 0.01–2.8	0	0	1 (0.3) CI: 0.05–1.7
Mercaptobenzothiazole (2.0% pet.)	1 (0.2) CI: 0.04–1.3	0	1 (0.4) CI: 0.08–2.4	0	1 (0.3) CI: 0.05–1.7
2‐Hydroxyethyl methacrylate (2.0% pet.)	1 (0.2) CI: 0.04–1.3	1 (0.5) CI: 0.01–2.8	0	0	1 (0.3) CI: 0.05–1.7
Total	185	75	110	52	133

**FIGURE 1:**
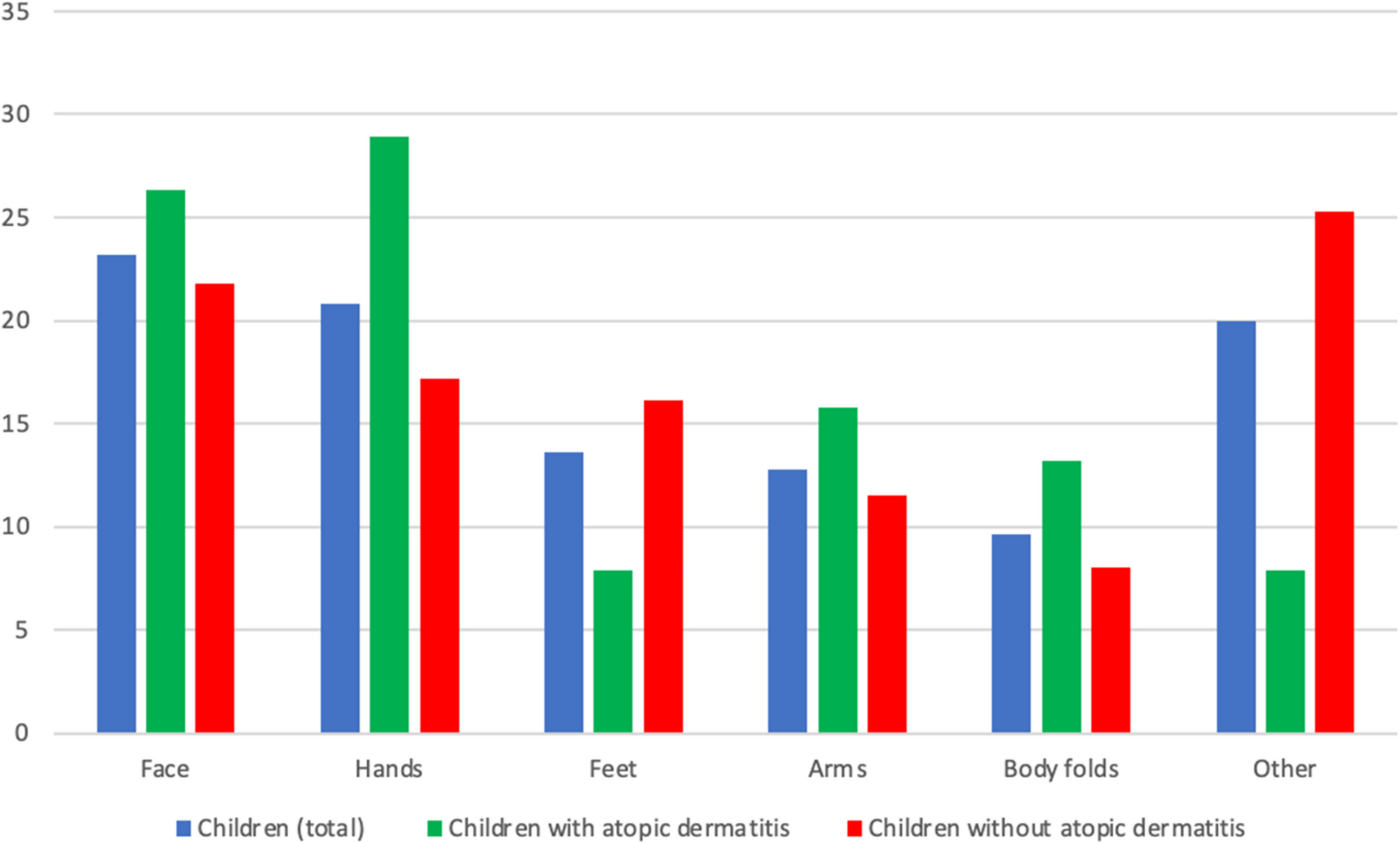
Sites involved in 125 children with at least one positive patch test reaction according to presence or absence of atopic dermatitis.

The most frequently involved sites in the 125 children with at least one positive patch test reaction were head (23.2%), hands (20.8%), feet (13.6%), arms (12.9%), and body folds (9.6%) (Figure 1). Face, hands, arms, and body folds were more frequently involved in children with AD than in those without AD (26.3% vs. 21.8%, 28.9% vs. 17.2%, 15.8% vs. 11.5%, 13.2% vs. 8.0%, respectively), while feet were mostly involved in children without AD (16.1% vs. 7.9%).

## 4 DISCUSSION

In the paediatric population, the prevalence of contact allergy is difficult to precise and the reported sensitivity rate in children largely ranges from 26.6% to 95.6%.^1^ This wide range is influenced by several factors, including diagnosis criteria for AD: most studies used the Hanifin and Rajka criteria, but in several studies, diagnostic criteria are not specified.^1^ Moreover, the upper age limit used in similar studies varies from 12^37^ to 18 years.^2^, ^3^, ^4^ Other factors such as clinical selection criteria for patch testing (selected and unselected populations), patch test series, and patch test methodology influence the children sensitivity rate.^1^ In the present study on 432 children aged up to 14 years, a prevalence of 28.9% was documented, lower than that reported by several other studies,^1^ probably due to low upper age limit of our patients. In fact, considering the five age groups, sensitivity rate ranged from 20.7% to 37.0% with an increasing trend according to age and consequently to longer allergen exposure,^4^ especially to toiletries, cosmetics, sport equipment, hair dyes, and jewellery.

According to previous studies,^1^, ^2^, ^38^ overall higher prevalence of positive patch test results was higher in girls (32.3%) than in boys (25.0%). Moreover, these data were observed especially in the first 2 age groups, where it resulted two times higher in girls than in boys (21/75, 28.0%, and 10/71, 14.1%, respectively). AD, present in 23.8% of tested children, seems to be a predisposing factor for contact sensitivity. In fact, we documented higher sensitization prevalence in children with AD (36.9%) than without AD (26.4%), probably due to the epidermal barrier impairment caused by the lack of filaggrin protein with T‐cell inflammation and the T‐helper 2 cell‐mediated pathways that worse damage of epidermal barrier.^39^ Literature data changed during the last decades, suggesting an increasing role of AD as risk factor for developing contact allergy. In fact, studies conducted until 2010 mostly showed a lower prevalence of contact allergy in children with AD.^1^ Studies performed in the following years reported an increasing prevalence in children with AD and recently, a higher prevalence of contact allergy in AD children population was documented, probably due to increased attention by dermatologists in AD diagnosis, a more frequent patch testing in refractory AD to investigate contact allergic component as potential aggravating factor, and increased use of cleansing and moisturizing products specifically formulated for AD^2^ (Table 3).

**TABLE 3:** Children with and without atopic dermatitis with at least one positive patch test reaction, according to recent literature data (2012–2022)

Study	Age range (years)	No. of children	Children with AD		Children without AD	
			No. of children (%)	No. of positive children (%)	No. of children (%)	No. of positive children (%)
Schena et al.^10^	0–15	349	123 (35.2)	68 (55.3)	226 (64.8)	174 (77.0)
Belloni Fortina et al.^8^	1–10	2614	1283 (49.1)	600 (46.8)	1331 (50.9)	631 (47.4)
Jacob et al.^14^	0–18	1117	552 (49.4)	337 (61.1)	565 (50.6)	499 (88.0)
Lubbes et al.^22^	0–18	921^a^	526 (52.0)	252 (47.9)	395 (48.0)	185 (46.8)
Romita et al.^18^	0–14	268	141 (52.6)	28 (19.9)	127 (47.4)	15 (11.8)
Noë et al.^2^	0–17	329	179 (54.4)	78 (43.6)	150 (45.6)	44 (27.3)
Total	0–18	5598	2804 (50.1)	1363 (48.6)	2794 (49.9)	1548 (55.4)

Abbreviation: AD, atopic dermatitis.

a This study included also 91 children with unknown atopic dermatitis status.

The most frequent sensitizers were metals (nickel sulphate, cobalt chloride, potassium dichromate), covering 45.9% of all 185 positive patch test reactions, followed by fragrances (14.6%) and isothiazolinones (13.5%). According to the current literature^1^, ^2^, ^4^ and disappointing the 2001 EU Nickel Directive,^40^ our results confirmed nickel sulphate as the most frequent contact allergen (10.2%), especially in girls (12.1%). This is probably due to the still wide diffusion of nickel‐containing products from non‐EU countries,^2^, ^38^ such as jewellery, toys, and electronics. Cobalt chloride, almost always as nickel sulphate co‐sensitivity (93.1%),^2^ is the second most common contact allergen (6.7%) being children exposed to metal‐plated products, crayons, and deodorants.^31^ We observed a prevalence of fragrance allergy similar to that of recent studies,^2^, ^4^ even if in our study a higher sensitivity rate for fragrance mix‐2 (3.2%) than fragrance mix‐1 (2.1%) was documented. The greater role of new fragrances than old fragrances as contact sensitizers in children, also recently observed by others,^41^ confirms the necessity to periodically re‐evaluate the fragrance mix composition according to EU cosmetic legislation.^42^ Moreover, the significant difference of positive patch test reactions to fragrance mix‐1 between children with and without AD is controversial in literature, confirming the findings of previous studies^22^, ^43^ and differing from others.^44^ Our data seem to be confirmed in adults with and without AD, although with a lower difference.^44^ Regarding isothiazolinones, MI prevalence (3.7%) was considerably and surprisingly higher than MCI/MI prevalence (2.1%), confirming that also in children, it is important to separately test MI at higher concentration to avoid false‐negative results.^45^, ^46^ Patch test concentration of MI (0.2%) was recently confirmed in children,^2^ demonstrating that the high prevalence observed by us is probably due to exposure to other than personal care products. The latter were regulated in 2014 by the European Commission Scientific Committee on Consumer Safety that banned MCI/MI from leave‐on products, allowing it in rinse‐off products not exceeding 1.5 ppm.^47^ In Italy, besides cosmetics, children are still exposed to declared and undeclared MI, such as toys, glue, slime, water‐based paint.^48^

Considering the eight most frequent positive allergens, all showed higher prevalence in children with than without AD, and in particular for fragrance mix‐1 (5.8% vs. 0.9%, *p* < 0.005), fragrance mix‐2 (5.8% vs. 2.4%), MI (5.8% vs. 3.0%), nickel sulphate (11.7% vs. 9.7%), potassium dichromate (3.9% vs. 2.4%), cobalt chloride (7.8% vs. 6.4%), and neomycin (2.9% vs. 1.5%). The possible presence of fragrances even in so‐called “hypoallergenic” daily used skin care products in the long‐term maintenance therapy of AD^28^ may explain the two highest prevalence differences of contact allergy to fragrance mix‐1 and fragrance mix‐2 (4.9% and 3.4%, respectively) between children with and without AD. These data could explain the most frequently involved sites of contact allergy in AD children such as hands, face, arms, and body folds, typical AD sites in children and adolescents. The higher prevalence rate of neomycin in AD children is due to the wide use in Italy of ointments based on aminoglycosides and in particular gentamycin, frequently cross‐reacting with neomycin.^49^

In conclusion, although the prevalence of contact allergy in children aged up to 14 years (28.9%) reported in this Italian study is consistent, these data are difficult to compare to that of other similar studies since age limits, selection criteria for patch testing, patch test series, and methodology are not uniform. Further studies with well‐standardized inclusion criteria are advisable to investigate the epidemiology and aetiology of contact allergy in children and to implement targeted secondary prevention strategies in this delicate patient age setting. We documented higher sensitivity rate in patients with (36.9%) than without AD (26.4%), in line with the most recent literature data. Among the baseline series allergens resulted most frequently positive to patch test, all of them were more frequently positive in children with than without AD, particularly for fragrance mix‐1 and fragrance mix‐2, MI, nickel sulphate, potassium dichromate, cobalt chloride, and neomycin sulphate. Therefore, in case of recalcitrant AD not responding to therapy, contact allergy needs to be investigated through patch test, the gold standard diagnostic tool also in paediatric population.

## CONFLICTS OF INTEREST

The authors declare that there are no conflicts of interest.

## Data Availability

The data that support the findings of this study are available from the corresponding author upon reasonable request.
